# Unilateral Horner’s Syndrome and Trigeminal Nerve Palsy After Lumbar Epidural Anaesthesia for Cesarean Section

**DOI:** 10.5152/TJAR.2021.923

**Published:** 2022-02-01

**Authors:** Ana Luísa Matos Vieira, Cândida Infante, Maria Costa, Ana Bernardino

**Affiliations:** Department of Anaesthesiology, Coimbra University Hospital Center, Coimbra, Portugal

**Keywords:** Cesarean section, epidural anaesthesia, Horner’s syndrome, trigeminal palsy

## Abstract

We report a healthy 29-year-old primigravida at 38 weeks gestation who underwent elective cesarean section and suffered from Horner’s syndrome and trigeminal palsy following epidural anaesthesia. The prompt recognition of this complication associated with lumbar epidural anaesthesia requiring close monitoring of the patient in order to prevent autonomic complications has been addressed.

**Main Points**

- Identifying and recognizing Horner’s Syndrome as a possible complication of epidural blocks, despite its low incidence.
- Importance of its early recognition and possible association with a high sympathetic blockade.
- Timely identification of its most common clinical signs could minimize the need for costly and inappropriate diagnostic investigation.

## Introduction

Horner’s syndrome (HS), which was first reported by Kepes et al^1^ in 1972, has been recognized as a rare and benign complication of lumbar epidural anaesthesia.

Horner’s syndrome results from a lesion of the sympathetic neurons that supply the head and neck. It is characterized by a classic triad of clinical signs: ipsilateral miosis, ptosis, and anhidrosis and is also commonly associated with enophtalmos and vasodilatation (conjunctival injection, facial flushing, and nasal obstruction).^2,3^ Paresthesia of the trigeminal nerve territory is a rare concomitant symptom associated with a high sensory block.^1,4^

We report HS and trigeminal nerve palsy after lumbar epidural anaesthesia in a parturient who underwent elective cesarean section. 

## Case Presentation

Otherwise healthy (ASA II class) 29-year-old primigravida at 38 weeks gestation was admitted for cesarean section (breech presentation of the fetus) under lumbar epidural anaesthesia. After obtaining informed consent of the patient in the operating room, standard monitoring was done and 500 mL Ringer’s lactate infusion was administered. Then, epidural anaesthesia was performed between L3/L4 intervertebral space via median approach in the left lateral decubitus position by inserting an 18G Tuohy needle using loss of resistance to air technique and a multiorifice epidural catheter (Braun Perifix^®^) citing in 4 cm. Epidural block was performed at first attempt uneventfully and a 15 mg of ropivacaine (Kabi®) was administered as a test dose.

Epidural catheter was functioning and 3 boluses were administered for a total volume of 7 mL of ropivacaine (55 mg) and sulfentanil (Hameln®) (10 µg). Fifteen minutes after epidural drug administration, the patient complained of heaviness in the right eyelid, hypoesthesia and numbness of the right hemiface, and paresthesia in the right hand. On physical examination, there was no change in her level of consciousness and her vital signs revealed arterial BP of 110/70 mm Hg, heart rate of 70 bpm, and normal breathing pattern with peripheral oxygen saturation of 98%-100%. 

**Figure 1.**
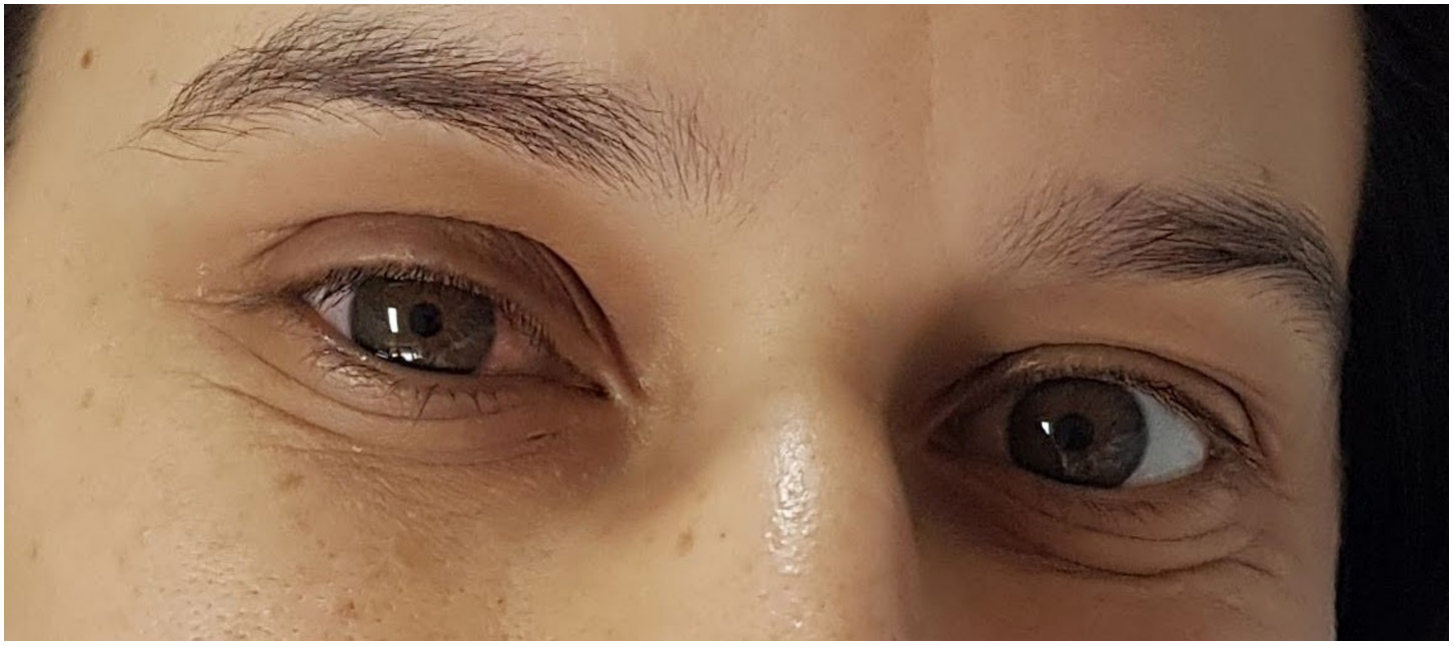
Right eye ptosis, miosis, and conjunctival congestion.

The sensory block level reached T1 on the right side and T4 on the left without motor block on the upper limb. The patient also showed right-sided ptosis, miosis, and conjunctival congestion, without any other visual symptoms or signs. She had paresthesia and reduced sensation to touch over her right face in the distribution of the ophtalmic and maxillary divisions of the trigeminal nerve and paresthesia in the right hand (Figure 1). 

Meanwhile, her epidural anaesthesia was adequate with a sensory block level of T4 for cesarean section and she delivered a healthy baby 20 minutes after epidural drug administration, without need for further top up. Vital signs were normal throughout the entire procedure.

The patient was monitored and symptoms gradually and spontaneously resolved completely over the following 18 hours. She was discharged after 72 hours with no neurologic sequela. The patient signed the consent form to publish the case and photos.

## Discussion

Although HS has been described as a rare complication after epidural anaesthesia in the obstetrics, recent literature review revealed 78 cases of HS in the context of prepartum epidural blocks until now.^2^ This syndrome is caused by disruption of the oculosympathetic pathway at the point where preganglionic neurons (second order) exit the spinal cord through the ventral roots on their path through the sympathetic chain to the superior cervical ganglion. These neurons have been shown to exit somewhere between C8 and T4.^5,6^

Several mechanisms have been proposed to explain the occurrence of HS after epidural anaesthesia. The cephalad spread of the local anaesthetic is the likely cause of HS in most cases, causing blockade of the sympathetic chain of neurons at C8-T1 level.^2,7^ Most reported cases of HS occurred in obstetric patients. This predilection may result from the anatomic and physiological changes that occur in pregnancy and favor the cephalad spread of the anaesthetic: narrowing of the epidural space as a result of distention of epidural veins; increased water content of connective tissues; partial occlusion of the inferior vena cava that diverts the blood through the epidural plexus venosus; and high progesterone levels increasing nervous fibers sensitivity to the local anaesthetic.^1,8,9^

Other neurologic signs that could appear as a result of a high block are hoarseness, respiratory difficulty, and haemo­dynamic instability, such as bradicardia and hypotension.^1,2,4^ Paresthesia of the trigeminal nerve territory can also be a concomitant symptom likewise in our case.^8,10^ The spinal trigeminal tract of the trigeminal nerve extends caudally until the second cervical segment, and the occurrence of trigeminal nerve palsy could be explained by high cephalad spread of the local anaesthetic.^4,11^

Other mechanisms that could further explain the appearance of HS are the patient’s position, the effects of gravity, and placing the catheter in a more cephalic position.^2^ There does not appear to be an increased likelihood of developing HS according to the type and concentration of anaesthetic agent used or methods of epidural anaesthetic delivery (bolus vs infusion).^2,12^

Additionally, accidental migration of the catheter to the subdural space might allow local anaesthetic to spread more cephalic dermatomes than expected.^8,13^ Because the subdural space extends beyond the foramen magnum and risk of subdural location of the catheter could explain trigeminal nerve involvement.^4,8^

In our patient, we observed high sensory blockade which occurred between C8 and T2 with right hand parestesia and while accompanying ipsilateral trigeminal nerve palsy without any spinal anaesthesia-induced hypotension. Also, sympathetic blockade and trigeminal nerve palsy were reported in 2 cases.^2^

## Conclusion

The present case report is unique because HS and trigeminal nerve palsy which are a rare composite complication of lumbar epidural anaesthesia for cesarean section have been revisited. They are most commonly self-limiting and disappeared when the block totally regressed, usually without neurologic sequela.
